# Search for carbapenem-resistant bacteria and carbapenem resistance genes along swine food chains in Central Italy

**DOI:** 10.1371/journal.pone.0296098

**Published:** 2024-01-05

**Authors:** Cristiana Garofalo, Cristiana Cesaro, Vesna Milanović, Luca Belleggia, Tullia Matricardi, Andrea Osimani, Lucia Aquilanti, Federica Cardinali, Giorgia Rampanti, Serena Simoni, Carla Vignaroli, Andrea Brenciani, Marina Pasquini, Maria Federica Trombetta

**Affiliations:** 1 Dipartimento di Scienze Agrarie, Alimentari ed Ambientali (D3A), Università Politecnica delle Marche, Ancona, Italy; 2 Dipartimento di Scienze della Vita e dell’Ambiente (DiSVA), Università Politecnica delle Marche, Ancona, Italy; 3 Dipartimento di Scienze Biomediche e Sanità Pubblica (DSBSP), Università Politecnica delle Marche, Ancona, Italy; Abadan University of Medical Sciences, ISLAMIC REPUBLIC OF IRAN

## Abstract

The presence of carbapenem–resistant bacteria and carbapenem resistance genes (CRGs) in livestock is increasing. To evaluate the presence of carbapenemase-producing Enterobacteriaceae (CPE) and the main CRGs along swine food chains of the Marche Region (Central Italy), samples of faeces, feed, and animal-food derived products were collected from seven small/medium, medium, and large-scale pig farms. A total of 191 samples were analysed using a culture-dependent method, with the aim of isolating CPE. Isolates were analysed for their resistance to carbapenems using a modified Hodge test and the microdilution method for the minimum inhibitory concentration (MIC) determination. Moreover, the extraction of microbial DNA from each sample was performed to directly detect selected CRGs via qPCR. Among the 164 presumptive resistant isolates, only one strain from a liver sample, identified as *Aeromonas veronii*, had an ertapenem MIC of 256 μg/mL and carried a carbapenemase- (*cphA*) and a β-lactamase- (*bla*_OXA-12_) encoding genes. A low incidence of CRGs was found; only nine and four faecal samples tested positive for *bla*_NDM-1_ and *bla*_OXA-48_, respectively. Overall, the importance of monitoring CPE and CRGs in livestock and their food chains should be stressed to control all potential non-human CPE and CRGs reservoirs and to determine safety levels for human health.

## 1. Introduction

Resistance to carbapenems represents a current major public health risk worldwide, as this class of antibiotics is used as the last therapeutic line of defence to treat human infections caused by multidrug-resistant Gram-negative bacteria [1–3]. The main bacterial mechanism of resistance to carbapenem is the production of carbapenemases, which are enzymes able to hydrolyse carbapenems and almost all β-lactam antibiotics, thus seriously limiting the therapeutic options to treat bacterial infections [4, 5]. This mechanism is worrisome, as the genes that code carbapenemases are frequently located on mobile genetic elements that are rapidly horizontally exchanged among commensal and pathogen bacteria, thus increasing their dissemination in a wide variety of bacterial species and reservoirs [3–14]. The most common plasmid-mediated carbapenemase-encoding genes (or carbapenem resistance genes–CRGs) include *bla*_KPC_, *bla*_NDM_, *bla*_VIM_, *bla*_OXA-48-_type, and *bla*_GES_; the primary bacterial members involved in spreading carbapenemase genes are ascribed to Enterobacteriaceae, *Pseudomonas* spp., and *Acinetobacter* spp. [2, 6, 8, 13–20]. The well-documented involvement of carbapenemase-producing Enterobacteriaceae (CPE) in community and health-care diseases associated with high mortality rates justifies the need for surveillance of CRGs and CPE and their spread into different environments [1, 4–6, 8, 13, 19, 21, 22]. It is also well-known that the massive use and misuse of antibiotics in humans, agriculture, aquaculture, and livestock in recent decades has led to selective pressure causing the rise of resistant bacteria [6]. Although carbapenems are banned for veterinary treatment worldwide [23–25], the detection of CPE and CRGs in animal husbandry is progressively increasing worldwide [6, 26–28]. It has been hypothesized that exposure to antimicrobials used in livestock, as third generation cephalosporins, may cause selection pressure favouring the development of cross-resistances to other antimicrobials that are commonly used in human therapy as carbapenems [6, 23, 29]. Livestock may act as a source of CPE and CRGs, which can be disseminated by faecal contamination of the environment and introduced into human digestive tracts via human diets if a cross-contamination of food products occurs (during the slaughter process, for instance) [29–33]. As reviewed by Bonardi and Pitino [6], the literature regarding the prevalence and transmission of CPE and CRGs in livestock remains inadequate and fragmented. Notwithstanding, swine and poultry are the most studied livestock in which Enterobacteriaceae and non-fermenting species (such as *Pseudomonas* spp. and *Acinetobacter* spp.) expressing carbapenem resistance have been most frequently detected [6]. Moreover, to the authors’ knowledge, to date few studies have focused on investigating carbapenem-resistant bacteria in pig livestock in Italy [34, 35], specifically in Central [7] and Northern Italy [36].

The present study aimed to draw a more complete picture of CPE occurrence using standard culture methods and the absolute quantification of *bla*_KPC_, *bla*_*OXA-48*_, *bla*_NDM_, *bla*_GES_, and *bla*_VIM_ genes using qPCR, along seven swine food chains in the Marche Region (Central Italy) to identify possible non-human reservoirs of CPE and CRGs, and to better define the role of animal-based products in the spread of such resistance via human diets.

## 2. Materials and methods

### 2.1. Sampling

Samples were collected from 7 small/medium, medium, and large-scale swine farms located in the Marche Region (Central Italy) between May 2019 and July 2021. The farms investigated do not benefit from the "antibiotic-free" certification. However, the survey shows that medicated feeds are used only until weaning in finishing-cycle farms. The pigs were reared in pens with paddocks and fed a commercial or self-produced diet based on barley, corn, and faba beans (S1 Table). All pigs were slaughtered at 160 kg mean live weight. The 100 g aliquots of raw pork meat, kidney, and liver samples (43 of each) were collected under sterile conditions at the selected farms’ slaughterhouses, immediately stored at 4°C, and processed within 24 h. To monitor the whole swine supply chain, 43 samples of faeces were also collected; for each sample approximately 60 g was obtained from a mix of 3–5 subsamples collected from healthy finishing pigs. Finally, 7 samples of feed were collected from the pig farms involved in the study, together with 12 samples of cured meats (salami, pork neck, bacon, cheek lard, loin, and soppressata-salami) that were collected from the same farms’ meat processing laboratories.

### 2.2. Detection of CPE using conventional methods

Aliquots of raw meat, kidney, liver, faeces, feed, and cured meat samples (~10 g) were homogenized in 90 mL of sterilized peptone water (2.0 g/L) for 2 min at 260 rpm using the Stomacher 400 circulator machine (International PBI, Milan, Italy). Next, 100 μL of each homogenate was inoculated onto MacConkey agar and incubated at 37°C for 24 h for the enumeration of Enterobacteriaceae. Parallelly, the same aliquot of each homogenate was inoculated in 10 mL of Luria–Bertani (LB) broth supplemented with 0.12 μg/mL ertapenem (Merck KGaA, Darmstadt, Germany) and incubated at 37°C for 24 h. Tubes showing turbidity were ten-fold serially diluted and 100 μL of the appropriate dilutions were spread on MacConkey agar containing ertapenem (0.12 μg/mL). After incubation at 37°C for 24 h, presumptive carbapenem-resistant colonies were selected and analysed for their carbapenemase activity using a modified Hodge test (MHT) [37] and for their resistance to carbapenems by minimum inhibitory concentration (MIC) determination, following the European Committee on Antimicrobial Susceptibility Testing (EUCAST) guidelines (https://www.eucast.org/ast_of_bacteria). MIC_50_ and MIC_90_ were defined as the MIC values able to inhibit the growth of 50% or 90% of isolates, respectively. Resistant isolates were evaluated for the class of carbapenemases they produced (KPC, VIM, and OXA_-48-_like types) through standard PCR using specific primers [38].

### 2.3. Whole Genome Sequencing (WGS) analysis

Only isolates resistant to ertapenem (via MIC determination and via the carbapenemase-production test, MHT), were identified to the species level using matrix-assisted laser desorption ionization time-of-flight (MALDI-TOF) (Bruker Daltonics, Bremen, Germany) and subjected to whole genome sequencing (WGS). WGS was performed by MicrobesNG Service (https://microbesng.com/) using the Illumina Miseq short-read technology (2 x 250 paired-end). The assembled and annotated draft genome provided by MicrobesNG Service was further analysed using free online bioinformatic tools at the Center for Genomic Epidemiology (CGE) (https://www.genomicepidemiology.org/). This Whole Genome Shotgun project has been deposited at DDBJ/ENA/GenBank under the accession JAULJM000000000.

### 2.4. DNA extraction and purification

A 1.5 mL aliquot of each sample homogenate (10^−1^ dilution) was processed for direct extraction of bacterial DNA using a commercial kit suitable to the sample type under examination. In detail, bacterial DNA from raw and cured meat, kidney, and liver was extracted using a PowerFood microbial DNA isolation kit (MoBio Laboratories, Carlsbad, California, USA); for animal faeces and feed samples an E.Z.N.A. soil DNA kit (Omega Bio-tek, Norcross, Georgia, USA) and DNeasy® PowerSoil® Pro kit (Qiagen, Hilden, Germany) were used, respectively, following the manufacturers’ instructions. DNA purity and yield were assessed spectrophotometrically (Nanodrop, Thermo Fisher Scientific, Waltham, MA, USA) and fluorometrically (Qubit, Thermo Fisher Scientific, Waltham, MA, USA).

### 2.5. qPCR

Absolute quantification of each carbapenemase gene (*bla*_NDM-1_, *bla*_VIM_, *bla*_GES_, *bla*_OXA-48-_, and *bla*_KPC_) in raw and cured meat, kidney, liver, faeces, and feed was carried out using the CFX Connect Real-Time PCR System (Bio-Rad, Hercules, California, USA) following the protocol previously described by Milanović et al. [12].

## 3. Results and discussion

**Fig 1:**
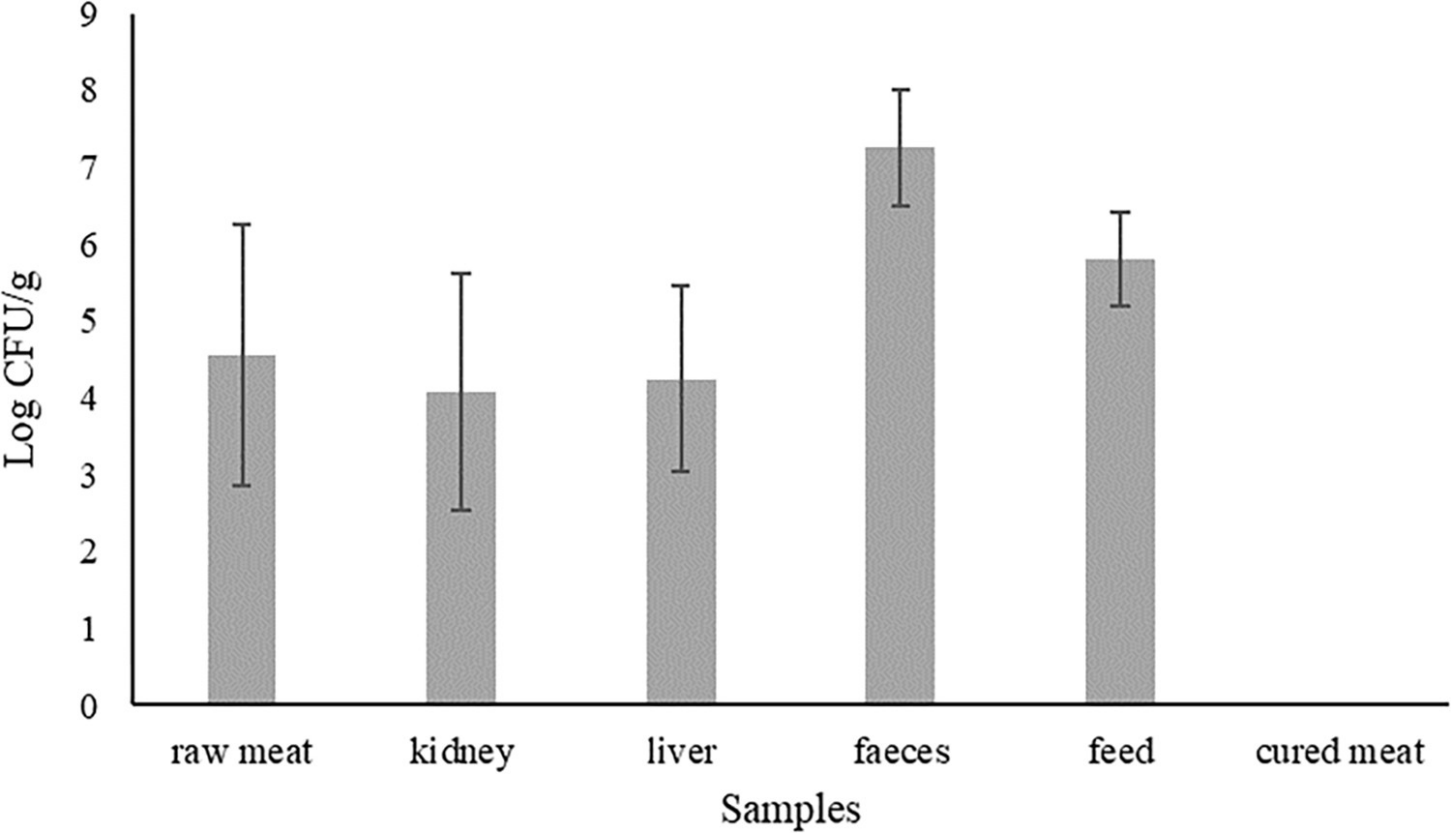
Mean values ± standard deviation of the viable counts obtained on MacConkey agar for each sample type.

All samples collected along the food chains of seven pig farms were screened for the presence of CPE and CRGs. Viable counts varied in different samples, as reported in Fig 1.

As expected, faecal samples had the highest Enterobacteriaceae loads, with a mean value of 7.25 Log CFU/g; these data were in line with those reported in the literature for finishing pig faeces and human faeces with between 5 and 9 Log CFU/g [39, 40]. Feed samples showed average counts of 5.80 Log CFU/g, which were comparable to those reported in the literature for hay (4–6 Log CFU/g) used as cattle feed [41] and for wheat (approximately 4–5.6 Log CFU/g) used as pig feedstock [42, 43]. Values of approximately 4 Log CFU/g, related to presumptive Enterobacteriaceae load, were registered in raw meat, liver, and kidney samples. These loads were probably due to Enterobacteriaceae contamination between meat and the environment or between meat and animals’ intestinal content during the slaughtering process. Indeed, the meat, and in particular the muscle, of slaughtered animals was sterile; however, during skinning and evisceration processes meat may contact the skin and/or the gastrointestinal tract content, which could lead to an increase in the bacterial load, specifically Enterobacteriaceae [44]. Therefore, great attention should be paid to these processes as key points for Enterobacteriaceae contamination [44]. The viable counts of Enterobacteriaceae were under the detection limit (< 1 Log CFU/g) of experimental conditions for some meat, kidney, and liver samples, as well as for all cured meat samples analysed in the present study, including salami, pork neck, bacon, cheek lard, loin, and soppressata-salami. For these processed and fermented products, Enterobacteriaceae presence and growth may have been inhibited by different factors, including the addition of salt and ripening, which reduce water activity, or by competition with other microorganisms added as starter cultures or naturally present in the raw material [45]. Therefore, the transformation processes justify the low number of Enterobacteriaceae.

Subsequently, after enrichment in broth containing ertapenem, 164 presumptive carbapenem–resistant isolates were collected from all sample types, except cured meat. However, MIC determination showed that all isolates, except one, were not resistant to carbapenems (Table 1). This result was related to the low concentration of ertapenem used for the primary screening of resistant isolates. However, we have not used concentration higher than 0,12 μg/mL not to exclude strains carrying resistance genes but phenotypically susceptible.

**Table 1:** Susceptibility to ertapenem of 164 isolates grown on MacConkey agar supplemented with ertapenem.

Sample type (total n = 191)	Number of presumptive resistant isolates (total n = 164)	Number of isolates positive to MHT^a^	MIC^b^ range (μg/mL)	MIC_50_^c^ (μg/mL)	MIC_90_^c^ (μg/mL)
**Faeces (n = 43)**	43	0	<0.25–1	0.25	1
**Raw meat (n = 43)**	36	0	<0.25–1	0.5	1
**Kidney (n = 43)**	37	0	<0.25–2	0.25	1
**Liver (n = 43)**	41	1	<0.25–256	0.5	1
**Cured meat (n = 12)**	0	-	-	-	-
**Feed (n = 7)**	7	0	<0.25–1	0.25	1

^a^MHT, Modified Hodge Test

^b^MIC, Minimum Inhibitory Concentration

^c^MIC_50_ and MIC_90_, the lowest concentration of the antibiotic at which 50 and 90% of the isolates were inhibited, respectively

**Fig 2:**
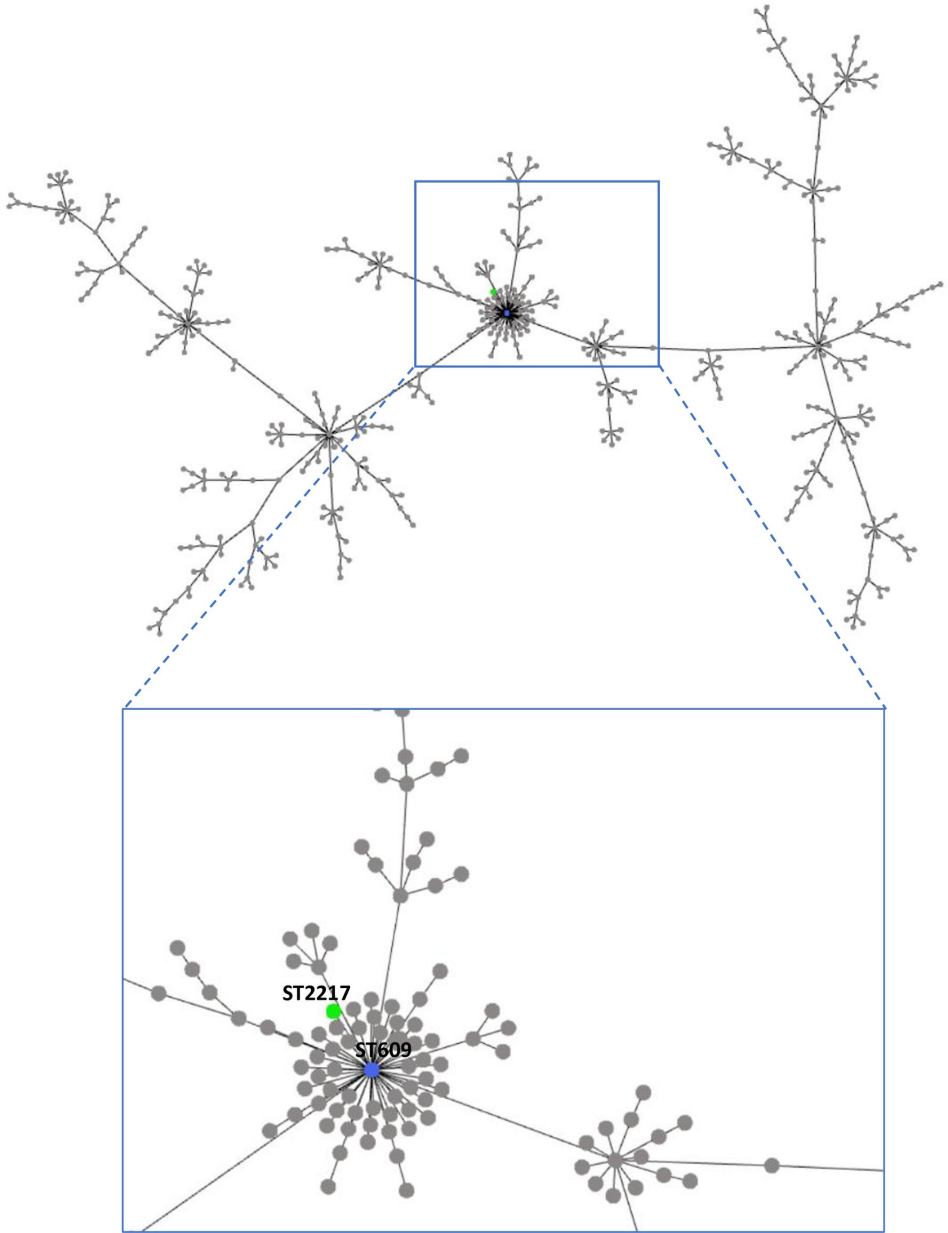
Phylogenetic tree created (https://online.phyloviz.net/) by multi-sequence alignment of six alleles’ FASTA input of all *Aeromonas veronii* isolates (n = 627) deposited in PubMLST. The *Aeromonas* strain of this study is identified by the green node. The ST609 (blue node) is also evidenced as the putative founder of the clonal complex in which our strain is included.

Only an isolate from a liver sample had a positive MHT result, with a 256 μg/mL MIC for ertapenem. The strain was also resistant to imipenem (4 μg/mL MIC), but susceptible to meropenem (1 μg/mL MIC). PCR assays confirmed the presence of a *bla*_OXA-48-_type gene encoding an OXA-48 family carbapenemase in the strain. The isolate was identified as an *Aeromonas veronii* by MALDI-TOF and its genome was completely sequenced. The assembled genome was submitted to the *Aeromonas* MLST database (https://pubmlst.org/bigsdb?db=pubmlst_aeromonas_isolates) to identify its sequence type (ST); it was assigned to the new ST2217, as sequences of all six loci were new alleles (*gltA*_1006, *groL*_917, *gyrB*_1003, *metG*_1047, *ppsA*_1159, and *recA*_1090). The phylogenetic tree of all *A*. *veronii* (n = 627) deposited in the PubMLST database is depicted in Fig 2.

The new ST2217 identified in this study showed phylogenetic correlations with the clonal complex formed by the ST609. According to the database (https://pubmlst.org/bigsdb?db=pubmlst_aeromonas_isolates), the ST609 corresponds to an *A*. *veronii* strain isolated in China from poultry in 2018. Although the strain was positive in PCR assays for *bla*_OXA-48_-type genes, WGS followed by ResFinder analysis demonstrated the presence of two beta-lactamase genes (*cphA* and *ampS*) located on the chromosome. The species-specific *cphA* gene [46], mainly found in *Aeromonas hydrophila*, *A*. *veronii*, and *Aeromonas jandae* isolates, encodes a metallo-β-lactamase active only on carbapenems. Our isolate carried a *cphA* showing 96% identity with the *cphA4* variant of an environmental *A*. *veronii* strain reported in the literature [47]. The *ampS* gene, encoding a class D β-lactamase, showed 99% coverage and 99% nucleotide identity with an OXA-912 enzyme included in the OXA-12 family, originally described in *Aeromonas sobria* [48].

*A*. *veronii* belong to the family Aeromonadaceae that contains members of the genus *Aeromonas*, which are Gram-negative rods and facultative anaerobes ubiquitous in nature, commonly isolated from water, soil, and food animals (shellfish, poultry, cattle, and pigs). Moreover, members of *Aeromonas* have been associated with a broad range of human infections (biliary tract infections, gastrointestinal tract syndromes, wound and soft tissue infections, and secondary bacteraemia) and several opportunistic diseases in animals [49–51]. Faecal carriage by healthy humans and animals has also been reported [50, 52, 53]. Thus, in the present study, the recovery of an *Aeromonas* strain in a single liver sample was likely correlated to faecal contamination of the sample during its handling. Previously, five strains of *A*. *veronii* harbouring the chromosomally encoded *bla*_ImiS_ were isolated from cattle faeces at a slaughterhouse in Switzerland [54]. Moreover, Zurfluh et al. [54] reported that this gene is generally co-transcribed with other β-lactamases located on chromosomes with overexpression induced by β-lactam or carbapenem antibiotics. Hence, using β-lactams in veterinary therapy could lead to selective pressure for naturally occurring chromosomal carbapenemases [1]. Therefore, using β-lactam antimicrobials to treat animals may be associated with the rise and dissemination of carbapenem–resistant *A*. *veronii* from animals to humans [54]. Among *Aeromonas* species, an overall increase in resistance to antibiotics, such as third generation cephalosporins, ampicillin, tetracycline, and ciprofloxacin (all commonly used/overused in clinical settings, agriculture, and fisheries) has been noted, thus suggesting that these bacteria could represent a potential environmental reservoir for antibiotic resistance genes [49, 51, 55]. Of note, food preservation techniques, such as cooling and vacuum packing, could promote *Aeromonas* spp. growth, as it has been reported that *Aeromonas* spp. have the capability to grow in anaerobic conditions at 4°C [54].

Regarding the qPCR analysis, efficiencies of 97.3%, 96.6%, 94.5%, 94.1%, and 93.15% were obtained for *bla*_OXA-48_, *bla*_KPC_, *bla*_NDM-1_, *bla*_VIM_, and *bla*_GES_ genes, respectively. The R^2^ values were >0.99, whereas the detection limit was <1 Log gene copies per reaction for all CRGs tested.

No CRGs were detected in raw and cured meat, kidney, liver, and feed samples; the faecal samples’ results are reported in Table 2. No samples were positive for *bla*_KPC_, *bla*_VIM_, or *bla*_GES_ genes; however, a very few faecal samples tested positive for *bla*_NDM-1_ and *bla*_OXA-48_ genes. In detail, *bla*_NDM-1_ was detected in 9 faecal samples (21%), ranging from 3.75 to 5.85 Log gene copies/g, with an average 5.04±0.83 Log gene copies/g, whereas *bla*_OXA-48_ was detected in the only 4 of 43 faecal samples (11%) with between 3.53 and 3.98 Log gene copies/g, with an average 3.75±0.19 Log gene copies/g.

**Table 2:** Results from qPCR for carbapenemase-encoding genes using DNA directly extracted from faecal samples.

		Carbapenemase resistant genes (gene copies/g ± standard deviation)				
Swine farm	Sample	*bla*_GES_	*bla*_KPC_	*bla*_OXA-48_	*bla*_NDM-1_	*bla*_VIM_
A	1	n.d.	n.d.	n.d.	n.d.	n.d.
	2	n.d.	n.d.	n.d.	n.d.	n.d.
	3	n.d.	n.d.	n.d.	3.97±0.07	n.d.
	4	n.d.	n.d.	n.d.	4.23±0.03	n.d.
	5	n.d.	n.d.	n.d.	3.75±0.06	n.d.
B	6	n.d.	n.d.	n.d.	n.d.	n.d.
	7	n.d.	n.d.	3.53±0.07	n.d.	n.d.
	8	n.d.	n.d.	3.67±0.05	n.d.	n.d.
	9	n.d.	n.d.	3.82±0.08	5.61±0.03	n.d.
	10	n.d.	n.d.	3.98±0.06	5.59±0.09	n.d.
	11	n.d.	n.d.	n.d.	n.d.	n.d.
	12	n.d.	n.d.	n.d.	n.d.	n.d.
	13	n.d.	n.d.	n.d.	n.d.	n.d.
	14	n.d.	n.d.	n.d.	n.d.	n.d.
	15	n.d.	n.d.	n.d.	n.d.	n.d.
C	16	n.d.	n.d.	n.d.	n.d.	n.d.
	17	n.d.	n.d.	n.d.	5.32±0.09	n.d.
	18	n.d.	n.d.	n.d.	5.21±0.06	n.d.
	19	n.d.	n.d.	n.d.	n.d.	n.d.
	20	n.d.	n.d.	n.d.	n.d.	n.d.
	21	n.d.	n.d.	n.d.	n.d.	n.d.
	22	n.d.	n.d.	n.d.	n.d.	n.d.
	23	n.d.	n.d.	n.d.	n.d.	n.d.
D	24	n.d.	n.d.	n.d.	5.85±0.06	n.d.
	25	n.d.	n.d.	n.d.	n.d.	n.d.
	26	n.d.	n.d.	n.d.	n.d.	n.d.
	27	n.d.	n.d.	n.d.	5.84±0.07	n.d.
	28	n.d.	n.d.	n.d.	n.d.	n.d.
E	29	n.d.	n.d.	n.d.	n.d.	n.d.
	30	n.d.	n.d.	n.d.	n.d.	n.d.
	31	n.d.	n.d.	n.d.	n.d.	n.d.
F	32	n.d.	n.d.	n.d.	n.d.	n.d.
	33	n.d.	n.d.	n.d.	n.d.	n.d.
G	34	n.d.	n.d.	n.d.	n.d.	n.d.
	35	n.d.	n.d.	n.d.	n.d.	n.d.
	36	n.d.	n.d.	n.d.	n.d.	n.d.
	37	n.d.	n.d.	n.d.	n.d.	n.d.
	38	n.d.	n.d.	n.d.	n.d.	n.d.
	39	n.d.	n.d.	n.d.	n.d.	n.d.
	40	n.d.	n.d.	n.d.	n.d.	n.d.
	41	n.d.	n.d.	n.d.	n.d.	n.d.
	42	n.d.	n.d.	n.d.	n.d.	n.d.
	43	n.d.	n.d.	n.d.	n.d.	n.d.
Average		n.dr.	n.dr	3.75±0.19	5.04±0.83	n.dr

n.d., not detected; n.dr., not determined

As recently reviewed by Bonardi and Pitino [6] and Hayer et al. [27], pig farms are among the most studied worldwide regarding carbapenem resistances assessment, as over 40% of meat consumed worldwide derives from domestic pigs that represent one of the main food-producing species [56]. Most of these studies are related to culture-dependant analysis aiming to isolate and identify CPE and non-Enterobacteriaceae-carrying carbapenemase genes. Although in the present study no CPE have been isolated, the culture-independent approach using qPCR demonstrated qPCR’s usefulness in detecting the presence of *bla*_NDM-1_ and *bla*_OXA-48_ genes. Both these genes encoded for common carbapenemases belonging to the plasmid-acquired class B NDM (New Delhi metallo-beta-lactamase, 10 variants) and class D serine-β-lactamases, including OXA carbapenemases (carbapenem-hydrolyzing oxacillinase) such as OXA-48 [12]. The overall prevalence of *bla*_NDM-1_ and *bla*_OXA-48_ (only detected in faecal samples) was low, accounting for 5% of all samples, thus indicating a low risk level concerning the diffusion of such resistances along swine food chains in our study area. Furthermore, the absence of CRGs within the meat and cured meat products under study confirmed a high safety level for consumers. Very recently, Hayer et al. [27] analysed the literature to identify the global distribution of CRGs in carbapenem-resistant or extended-spectrum β-lactamase (ESBL)-producing *Escherichia coli* isolates in pigs; they reported that *bla*_NDM-1_ and *bla*_OXA-48_ have been widely distributed with low frequency, mainly in Asian countries such as China, India, and South Korea, since 2001. Concerning Europe, as was found in the present study, CPE were absent from German pig farms, whereas *bla*_OXA-48_ has been detected in 1 of 318 analysed stool samples [57]. In addition, *bla*_OXA-48_ was harboured by two *E*. *coli* isolates from faecal samples from an Italian pig farm in coexistence with *bla*_OXA-181_, and was located on a 51.5-kb non-conjugative plasmid [35]. In contrast, Carelli et al. [7] reported a high frequency of *bla*_OXA-48-_type genes in faeces (72.5%) and meat (26%) of livestock animals (pigs and cattle) within the Marche Region identified using Droplet Digital PCR (ddPCR). This result could be explained by different detection methods, PCR protocols, and primers used that spanned all OXA-48-related variants, including OXA-48 and OXA-181, as well as different samples, including cattle in addition to pigs. Therefore, developing a stable monitoring system with sensitive and harmonized CPE- and CRGs-detection programmes for livestock and food products is of great importance to detect both bacteria and genes responsible for carbapenem-resistances, and to prevent their increased diffusion into the environment and subsequently into human food chains.

## 4. Conclusions

Monitoring CPE and CRGs occurrence in pig livestock using culturable and sensitive molecular methods is crucial to identify potential reservoirs of transferable carbapenems resistances and to try to avoid the active transmission of CPE and CRGs from animals to humans through diet. Despite the absence of CPE isolates and the low overall CRGs prevalence at pig farms sampled during 2019–2021 within the Marche Region in Italy, it is necessary to intensify livestock carbapenem resistance surveillance programs, as underlined by the EFSA BIOHAZ Panel [1], as well as to apply a prudent use of antibiotics and control measures to prevent the spread of carbapenemase-producing bacteria in food-producing animals and the human food chain.

## Supporting information

S1 Table Details of pig Italian farms involved in the study. (DOCX)
