# Supplementary material for: Search for carbapenem-resistant bacteria and carbapenem resistance genes along swine food chains in Central Italy
Source: PLoS One. 2024 Jan 5;19(1):e0296098. doi: 10.1371/journal.pone.0296098 (PMC10769077; doi:10.1371/journal.pone.0296098)
Supplement: S1 Table — (DOCX) [file pone.0296098.s001.docx]

**Supplementary Table 1.** Details of pig Italian farms involved in the study.

| **Farm ID** | **Farm Location**  **(Province)** | **Size^1^** | **Slaughtered Pigs/year**  **(# Heads)** | **Other**  **Species**  **(# Heads)** | **Farming**  **Type^2^** | **Finishing**  **Feeding^3^** | **Piglets**  **Feeding^3^** | **Grain crop**  **area**  **(# Ha)** | **Pasture**  **area**  **(# Ha)** |
| --- | --- | --- | --- | --- | --- | --- | --- | --- | --- |
| **1** | Hills  (Pesaro-PU) | Medium | 80 | Cattle (80) | F-c | S-p | C | 99 | 25 |
| **2** | Hills  (Fabriano-AN) | Medium | 100 | Cattle (70) | C-c | S-p | S-p | 90 | 10 |
| **3** | Hills  (Fabriano-AN) | Medium | 95-100 | Cattle (15)  Goats (5)  Poultry (100) | C-c | S-p | C | 60 | --- |
| **4** | Hills  (Macerata-MC) | Large | 6.000 | --- | C-c | S-p | S-p | 50 | 5-6 |
| **5** | Hills  (Montegiorgio-FM) | Large | 2.000 | --- | C-c | C | C | 100 | --- |
| **6** | Hills  (Rapagnano-FM) | Large | 25.000 | --- | C-c  F-c | C | C | 170 | --- |
| **7** | Hills  (Recanati-MC) | Small/medium | 45 | Cattle (45) | C-c | S-p | C | 30 | --- |

^1^ Small = 1-30 slaughtered pigs/year; Small/medium = 31-50 slaughtered pigs/year; Medium = 51-250 slaughtered pigs/year; Medium/large =51-500 slaughtered pigs/year; Large = ≥ 501slaughtered pigs/year.

^2^ Closed-cycle = C-c; Finishing-cycle = F-c.

^3^ Commercial = C; Self-produced = S-Pp.
